# Renal function during rofecoxib therapy in patients with metastatic cancer: retrospective analysis of a prospective phase II trial

**DOI:** 10.1186/1756-0500-4-2

**Published:** 2011-01-05

**Authors:** Stephan W Reinhold, Albrecht Reichle, Sonja Leiminger, Tobias Bergler, Ute Hoffmann, Bernd Krüger, Bernhard Banas, Bernhard K Krämer

**Affiliations:** 1 Klinik und Poliklinik für Innere Medizin II, University of Regensburg, Franz-Josef-Strauß-Allee 11, Regensburg, 93053, Germany; 2 Abteilung für Hämatologie und Internistische Onkologie, University of Regensburg, Franz-Josef-Strauß-Allee 11, Regensburg, 93053, Germany; 3 V. Medizinische Klinik, Universitätsklinikum Mannheim, University of Heidelberg, Theodor-Kutzer-Ufer 1-3, Mannheim, 68167, Germany

## Abstract

**Background:**

Angiostatic/antiinflammatory therapy with COX-II inhibitors and pioglitazone seems to be a well tolerated and promising regimen in patients with metastatic cancer. COX-II inhibitors may have less gastrointestinal side effects than conventional non-steroidal antiinflammatory drugs, but their impact on renal function seems to be similar.

**Methods:**

87 patients with metastatic/advanced cancer were treated up to 12 months (mean 19.5 weeks) with rofecoxib, pioglitazone and either capecitabine (group A with gastrointestinal and urological cancer, n = 50) or trofosfamide (group B with non-gastrointestinal/non-urological cancer, n = 37) and followed for further 6 months.

**Results:**

Baseline serum creatinine concentration was 0.81 ± 0.28 mg/dl, and increased by about 0.15 mg/dl during months 1-3. Accordingly estimated glomerular filtration rate (eGFR) decreased from 90.3 ml/min ± 3.6 ml/min at baseline by about 10 ml/min during months 1-3. Renal function decreased in 75 patients (86%) in the first month (p < 0.0001). This decrease went along with clinical signs of volume expansion. Renal function tended to recover after discontinuation of the study medication.

**Conclusions:**

Therapy with rofecoxib in an antiangiogenic/antiinflammatory setting results in a decrease of renal function in nearly every patient.

**Trial registration number:**

German Clinical Trials Register DRKS: DRKS00000119

## Background

Cyclooxygenases (both isoforms, COX-I and COX-II) oxidize arachidonic acid to prostaglandin H2, which is converted by different synthases to prostaglandin-E2, -D2, -I2, -F2α, and thromboxane A2. These prostaglandins inhibit apoptosis and promote cell division, metastasis and angiogenesis leading to increased tumor growth [1]. An antiangiogenic/antiinflammatory therapy with COX-II inhibitors and pioglitazone combined with metronomic low-dose chemotherapy with either capecitabine or trofosfamide seems to be well tolerated and promising in patients with advanced carcinomas [2,3]. However, in one study in patients with colorectal cancer increased gastrointestinal toxicity was reported [4]. Since COX-II inhibitors are known to elicit renal side effects to a similar extent than conventional non-steroidal antiinflammatory drugs [5], the detailed analysis of any change in serum creatinine concentrations and glomerular filtration rate in a prospective trial of antiangiogenic/antiinflammatory therapy in advanced cancer was our primary objective.

## Methods

### Patient Characteristics

The study was approved by the local ethics committee, and all patients gave their written informed consent for study participation. Patients with either gastrointestinal/urological cancer (group A, see Table 1) or with non-gastrointestinal/non-urological cancer (group B, see Table 1) were included in the study and treated with rofecoxib, pioglitazone and either capecitabine in a dose of 1.0 g bid (group A) or trofosfamide 50 mg tid (group B). Additional eligibility criteria have already been published [3].

**Table 1:** Baseline patient characteristics and underlying malignancy

Age in years (range)	60.5	(30 - 81)
Gender		
Male	56	(64%)
Female	31	(36%)
Baseline serum creatinine concentration in mg/dl (± SEM)	0.81	(± 0.28)
Arterial hypertension	21	(24%)
		
Concomitant nephrotoxic/volume depleting or blood pressure lowering medication		
- NSAIDs	18 (21%)	
- Loop diuretics	14 (16%)	
- Thiazides	8 (9%)	
- ACE inhibitor	6 (7%)	
- AT2 blocker	4 (5%)	
- Aldosterone antagonists	2 (2%)	
		
Preceding chemotherapy	45 (52%)	
Liver metastasis	45 (52%)	
	**Group A (treated with capezitabine, rofecoxib and pioglitazone)**	**Group B (treated with trofosfamid, rofecoxib and pioglitazone)**

Melanoma		16
Gastric carcinoma	13	
Colorectal carcinoma	12	
Renal cell carcinoma	9	
Hepatic carcinoma/Klatskin	7	
Sarcoma		5
Pulmonary adenocarcinoma/SCLC		3
Pancreatic cancer	3	
Urothelium carcinoma	3	
Gall bladder carcinoma	3	
Breast cancer		2
Histiozytosis X		2
Hodgkin/Non-Hodgkin-Lymphoma		2
Ovarial carcinoma		2
TNE		2
Chronic lymphatic leukemia		1
Cervix carcinoma		1

SCLC = small cell lung cancer; TNE = throat nose ear

Patients were recruited between 2000 and 2004. Treatment was administered up to 12 months and patients were followed up for further 6 months. 25 mg rofecoxib/day was administered to 75 patients, and a reduced dose (12.5 mg/day) was administered to 12 patients with pre-existing renal impairment. Renal impairment, that triggered a dose reduction of rofecoxib in the prospective phase II trial, had been defined as any serum creatinine concentration that exceeded the normal range. Serum creatinine concentrations were measured before inclusion of a patient and every month thereafter. The normal range of serum creatinine concentration in our institution is 05.-0.8 mg/dl for women and 0.8-1.1 mg/dl for men.

Glomerular filtration rate was estimated using the method of Cockcroft and Gault [6].

Provisions were made to reduce rofecoxib dose in case of a rise in serum creatinine concentration above 1.3 mg/dl or WHO grade >1 edema.

The study was stopped because of withdrawal of rofecoxib from the market.

### Statistics

We used the Wilcoxon signed rank test for the retrospective data analysis. Data are given as mean ± standard error of the mean.

## Results

### Serum creatinine concentration and glomerular filtration rate

**Figure 1:**
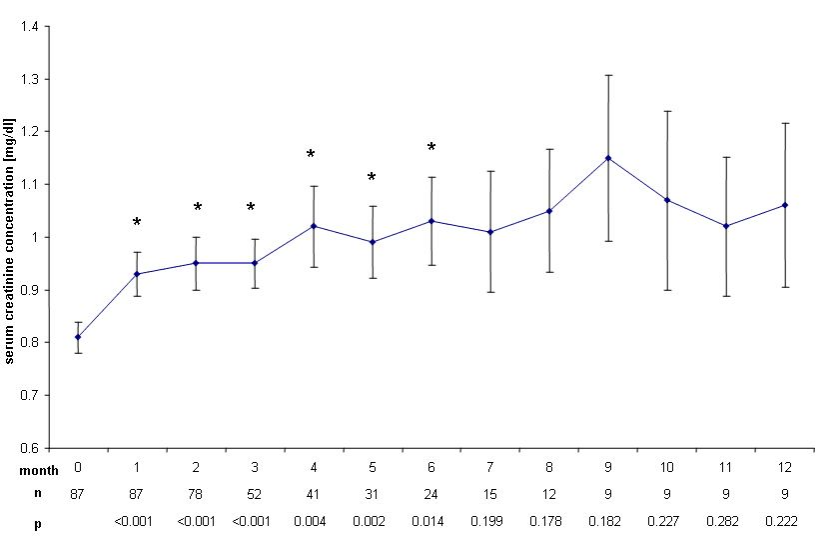
**Change of serum creatinine concentrations during therapy**. Increase of serum creatinine concentrations ± SEM compared to baseline (0.81, ± 0.28 mg/dl) during therapy with rofecoxib. *: p < 0.05.

**Figure 2:**
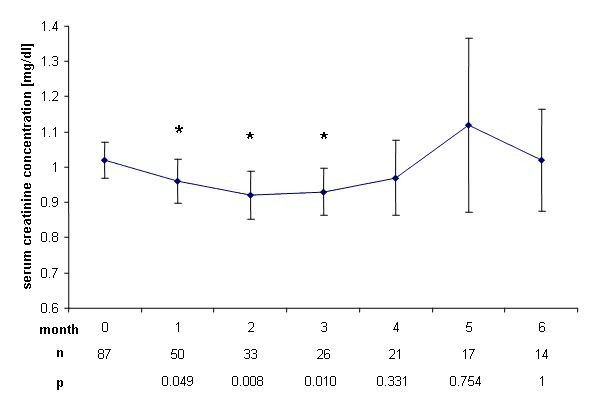
**Change of serum creatinine concentrations after end of therapy**. Decrease of mean serum creatinine concentrations ± SEM after end of therapy with rofecoxib compared to the last day of treatment. *: p < 0.05.

Serum creatinine concentration increased in 75 patients (86%) during the first month of the treatment phase (p < 0.0001). This significant increase was sustained during the first six months (Figure 1). After the end of treatment, creatinine concentration significantly decreased during months 1-3, but did not reach baseline concentrations again (Figure 2).

**Figure 3:**
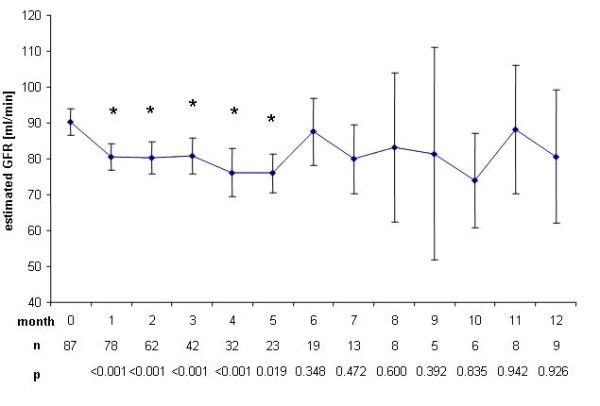
**Change of glomerular filtration rate during therapy**. Decrease of estimated glomerular filtration rate ± SEM compared to baseline (90.3 ml/min) during therapy with rofecoxib. *: p < 0.05.

**Figure 4:**
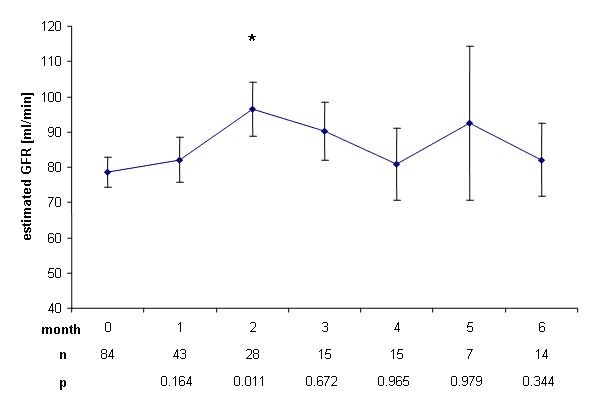
**Change of glomerular filtration rate after end of therapy**. Increase of estimated glomerular filtration rate ± SEM after end of therapy with rofecoxib compared to the last day of treatment. *: p < 0.05.

Mean baseline eGFR decreased accordingly from 90.3 ml/min ± 3.6 ml/min at baseline to a minimum of 76 ml/min ± 5.4 ml/min in the fifth month (p = 0.0195) (Figure 3). Two months after end of treatment with rofecoxib, mean eGFR reached baseline values again (96.6 ml/min ± 7.6 ml/min) (Figure 4).

In patients with pre-existing renal impairment, treated with a reduced dose of rofecoxib the average serum creatinine concentration rose from 1.01 mg/dl ± 0.1 mg/dl to a maximum of 1.25 mg/dl ± 0.27 mg/dl in the fifth month (p = 0.0058).

### Serum sodium concentrations

Serum sodium concentrations during rofecoxib therapy remained unchanged. A decrease from 139.8 mmol/l ± 0.39 mmol/l to 137.0 mmol/l ± 1.28 mmol/l in month nine (p = 0.006) appears to be a chance finding due to low numbers of patients remaining in the study. Serum sodium concentration remained stable after treatment stop.

### Body weight and edema formation

Mean body weight before treatment was 73.7 kg ± 1.7 kg, fluctuated from month to month with a lower mean body weight on month 2, 5 and 7, but increased significantly during therapy with rofecoxib up to the eighth month with 10 patients remaining at risk. The highest increase in mean body weight of 4.1 kg was noted in the eleventh month after start of rofecoxib treatment with only 8 patients remaining in the study. The increase in body weight went along with edema formation in 30 patients (34%).

Immediately after discontinuation of treatment mean body weight fell significantly from 74.2 kg ± 1.7 kg to 72.1 kg ± 2.5 kg.

### Blood pressure

Mean systolic blood pressure rose during therapy from 130.1 mmHg ± 2.1 mmHg to 137.8 mmHg ± 6.2 mmHg after twelve months except in months 2, 6, 7 and 10, where the mean systolic blood pressure was at or lower than baseline. In the first, second and fourth month after cessation of rofecoxib therapy, mean systolic blood pressure was significantly lower than during therapy.

Mean diastolic blood pressure remained unchanged throughout the trial.

## Discussion

We found a significant increase in mean serum creatinine concentration (p < 0.0001) already in the first month after initiation of treatment with rofecoxib. This increase was sustained during treatment and ranged around 0.15 mg/dl, corresponding to a decrease of eGFR of around 10 ml/min, reaching up to 14 ml/min in month 4. However, mean serum creatinine concentration remained within the normal range during and after end of the trial and no dialysis treatment was needed. Rofecoxib is known to cause acute renal failure at a rate of 1.2 to 1.5% in trials studying several thousands of patients [7,8], but even a single dose of rofecoxib can cause severe acute renal failure [9]. Furthermore, the half life of COX-II inhibitors rofecoxib and celecoxib is prolonged in liver failure [10]. Though patients with severe hepatic failure were excluded from the present study, 52% of the patients presented with liver metastasis and thus might have been subjected to a higher exposure of rofecoxib due to prolonged half life. Progressive underlying malignant disease may as well have contributed to functional prerenal failure i.e. due to decreased fluid intake. The decreased renal function however tended to normalize within 2 months after cessation of rofecoxib therapy (Figure 2 and 4), suggesting rofecoxib as the more likely cause of decreased renal function, especially as we observed no severe nausea, vomiting or diarrhea during the treatment phase.

We observed edema formation in 30 patients (34%), which is above the rate observed in large studies ranging between 7.7 and 23.3% [8,11,12] and which is possibly due to the persistent decrease in renal function, the well-known sodium- and/or fluid retaining properties of COX-2 inhibitors and thiazolidindiones, and the underlying malignant disease with hypoproteinemia.

The rise in mean systolic blood pressure during therapy corresponds to published data [13,14] and went along with increasing body weight, probably both reflecting renal impairment and subsequent fluid retention. Weight gain is often seen in patients with diabetes treated with pioglitazone [15] and pioglitazone may have contributed to edema formation and the observed increase in body weight in our study, but weight gain in our study population is even more astonishing in regard of the underlying malignancy which usually leads to progressive weight loss as demonstrated after end of treatment.

## Limitations

The antiinflammatory antiangiogenic therapy with a COX-II inhibitor in combination with pioglitazone and either trofosfamide or capecitabine seems to be both well tolerated and promising with primary disease response rates reaching up to 19% [3].

This study was stopped because of withdrawal of rofecoxib from the market, but the concept of an antiinflammatory antiangiogenic therapy with COX-II inhibitors proves to be true also for other COX-II inhibitors like celecoxib, still available on the market [16-18]. There are also clearly class effects like prolonged half life in liver failure [10] or decreasing renal function, which have been shown for rofecoxib as well as for celecoxib [19].

The decrease in renal function in our study has to be seen in the clinical context. A rise in mean serum creatinine concentration of 0.15 mg/dl is of limited importance to young patients with normal body weight and normal renal function. However, a mean fall in eGFR of up to 14 ml/min nevertheless may urge the physician to adapt the dose of concomitant medications or even stop the treatment with a COX-II inhibitor especially in the elderly patient with reduced body weight and pre-existing renal failure, suggesting the report of our results to be of persistent importance even in the light of withdrawal of rofecoxib from the market.

## Conclusion

We recommend close monitoring of renal function and blood pressure of patients with cancer treated with COX-II inhibitors, especially in a setting with pre-existing renal and hepatic failure.

## Competing interests

The authors declare that they have no competing interests.

## Authors' contributions

AR and BKK shared the conception and design of the study. SWR, UH, AR, BKK and SL were involved in data acquisition. SWR, BK, UH, BB and BKK were involved in statistical analysis and interpretation of the data. SWR, TB, BK, BB, AR and BKK participated in drafting and revision of the manuscript. All authors read and approved the final version of the manuscript.
